# Hydro-morphodynamic numerical modeling indicates risk zones for riverbed clogging

**DOI:** 10.1038/s41598-025-95150-3

**Published:** 2025-03-29

**Authors:** Federica Scolari, Mohammed Fadul, Sebastian Schwindt

**Affiliations:** 1 Department of Hydraulic Engineering and Water Resources Management, University of Stuttgart, 70569 Stuttgart, Germany; 2 Wasserversorgung Sulinger Land, 27232 Sulingen, Germany

**Keywords:** Large wood, Fine sediment, Suspended load, Vertical connectivity, Numerical simulation, MultiPAC, Ecology, Hydrology, Engineering, Mathematics and computing

## Abstract

Riverbed clogging compromises the ecological functioning of gravel-bed rivers. Physical clogging affects aquatic habitats and occurs when fine sediments infiltrate coarser substrates, reducing permeability, porosity, and oxygenation. Clogging analyses mostly rely on methods or models that assess the clogging state from field data without predictive capacity. However, predictive tools are essential to optimize habitat restoration in mountain rivers. This study evaluates a two-dimensional numerical model for simulating fine sediment infiltration and mobilization around two large wood pieces in a fully controlled morphodynamic gravel-bed channel. Field data from the morphodynamic channel, collected before and after an artificial flood, were used to calibrate the model and compare its outputs with measured sediment parameters. The model reproduces fine sediment fractions in the surface and subsurface layers, especially in a shallow, high-velocity zone that underwent substantial declogging. In contrast, fine sediment fractions increased in a deeper, slower-flowing zone. Additionally, simulated suspended sediment concentration and fine sediment fraction maps highlight how a swale saturated with fine sediment on the floodplain contributed to increased fine sediment infiltration and clogging downstream. These findings demonstrate that a robust 2d model capturing fine sediment dynamics can effectively identify clogging-prone areas in gravel-bed rivers.

## Introduction

Riverbed clogging, a critical process affecting river health and ecological function, remains difficult to predict accurately with existing modeling approaches. Clogging involves the infiltration and accumulation of fine sediments in the pore space of coarser riverbeds, reducing permeability and altering ecological conditions^1–4^. Gravel to cobble bed rivers, characterized by bed material with a mean coarse particle size from 2 to $256~10^{-3}$ m^5^, are particularly prone to clogging when their coarse sediment supply shifts toward finer sediment. Land use changes can artificially drive this shift because fine sediment erodes more readily from agricultural soils than coarse sediment from undisturbed surfaces^6,7^. Dams also promote clogging by retaining coarse fractions while allowing fine, suspended fractions to pass through their outlets, depriving downstream reaches of coarser sediment^8–10^.

Clogging mechanisms can be divided into biological and physical components. Biological clogging arises when organic matter, such as algae or microbial biofilms, accumulates on or within the riverbed substrate, limiting permeability and changing habitat conditions^11^. Physical clogging is further categorized into inner clogging, where fine sediment accumulates in subsurface layers, and outer clogging, where a fine sediment layer forms on the surface, impeding infiltration and affecting surface-dwelling organisms^2,4^. Inner clogging is driven by processes such as sieving and percolation during morphologically effective floods that carry mainly fine sediment^12,13^. Outer clogging occurs if calm water zones with low shear stress accumulate sediment on the bed surface^1,2,13,14^.

From an ecohydraulic perspective, understanding and forecasting riverbed clogging is essential for managing aquatic habitat quality. Conventional field^2^ and laboratory^15^ assessments measure clogging in localized settings using methods such as the colmameter^16^ or a multi-parameter approach known as MultiPAC^3,17^, which quantifies relevant attributes including grain size distribution, porosity, hydraulic conductivity, and dissolved interstitial oxygen^1,18,19^. While these approaches provide detailed local data, their spatial coverage and predictive capacity are limited. On the contrary, numerical models calibrated with field data represent an approach to forecasting hydraulic system behavior. The integration of hydraulic solvers and sediment transport modules is well-established in many modeling frameworks^20^, which demonstrated the coupling of these elements to simulate sediment transport and bed evolution. Many models also include vertical sediment exchange processes, such as oxygen infiltration and changes in hydraulic conductivity, which are critical for evaluating riverbed clogging^21–25^. However, while these models can simulate sediment transport dynamics and bed evolution, they are not typically used to evaluate the clogging of riverbeds specifically. In contrast, this study focuses on the accumulation and removal of fine sediment from the riverbed as a proxy for assessing clogging phenomena, offering a novel approach to simulating the fine sediment dynamics that drive inner and outer clogging, which cannot be easily assessed using traditional methods alone.

Two-dimensional (2d) hydro-morphodynamic numerical models, incorporating sediment transport equations and flow-sediment interactions, have the potential to capture fine sediment deposition and infiltration processes that contribute to physical clogging. However, morphodynamic simulations remain challenging due to the complexity of modeling multiple modes of sediment transport (suspended load and bedload) and the associated changes in bed topography caused by sediment deposition and erosion^26–28^. Although advances in computational capacity and numerical software have improved stability, such simulations remain demanding. Thus, there is a growing incentive to explore whether hydro-morphodynamic modeling can yield predictions of clogging dynamics, especially with regard to fine sediments, rather than merely post-event descriptions. Therefore, this study posed the question of whether a hydro-morphodynamic 2d-numerical approach can predict key indicators of clogging or declogging. The test hypothesis stated that the evolution of fine sediment fractions (FSFs) in the surface and subsurface layers of a gravel-bed river can be modeled and reproduced accurately. Although FSF alone cannot fully describe riverbed clogging, fine sediment infiltration into coarse riverbeds is a main driver of inner and outer clogging, causing reduced interstitial oxygen and hydraulic conductivity^2–4,17^. To examine this hypothesis, the open TELEMAC software suite^29^ was used in this study to simulate an artificial flood experiment at a morphodynamic fishway.

## Methods

### Field site and available data

**Fig. 1:**
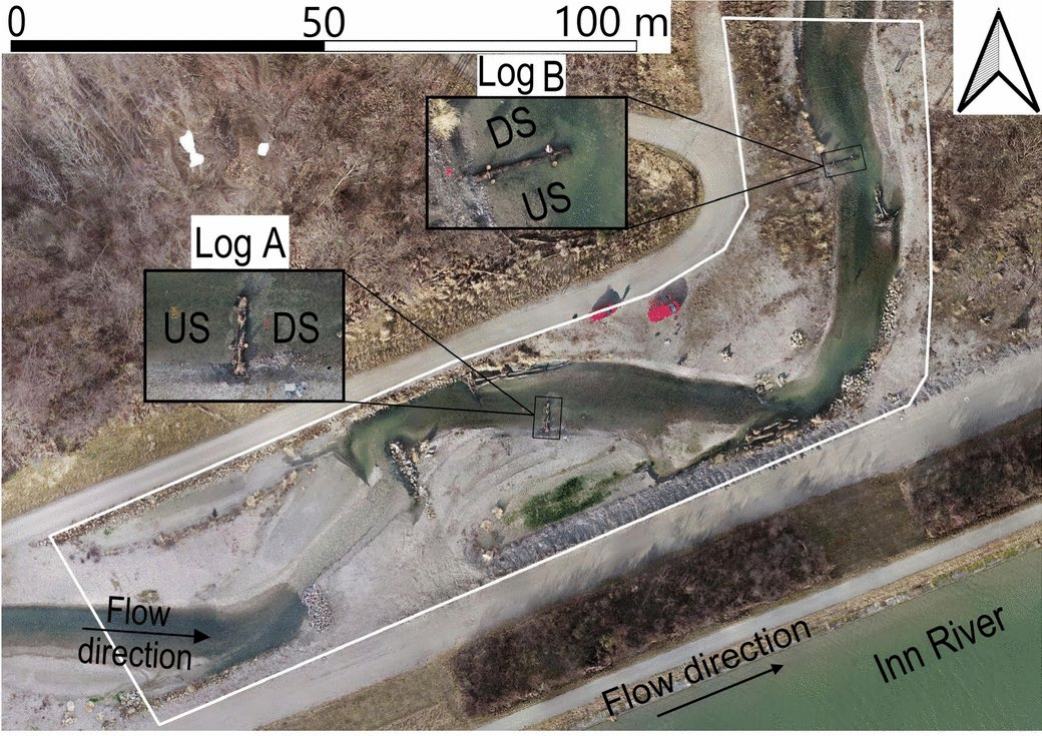
The study site of the fishway around the Ering–Frauenstein hydropower plant at the Inn River (Germany), indicating the location of two large wood pieces (log A and log B) to trigger declogging.

The study was conducted on a morphodynamic fishway bypassing the Ering–Frauenstein hydropower plant on the lower Inn River in Germany. The fishway extends about 2.6 km and exhibits a near-natural morphology with alternating deep and shallow sections, gravel patches, and pools (Fig. 1). The channel exhibits a longitudinal slope of about 4.7‰ in its upper part, which transitions into a gentler slope of 1.1‰ in a downstream section surrounded by alluvial forests. This study was implemented at the end of the upstream section. The fishway spans a total elevation difference of approximately 10 m. Its baseflow of $2\,\hbox {m}^{3}{s}^{-1}$ can be increased to a maximum of $12~\hbox {m}^{3}{s}^{-1}$ during flushing operations through an intake structure^30^. Designed to mimic a near-natural channel, the fishway provides multiple aquatic species with an alternative route that bypasses the hydropower facility, while also offering spawning, feeding, and refuge habitats. It further contributes to reconnection efforts aimed at linking the main river with adjacent floodplains. The re-discovery of the Danube gudgeon, previously presumed extinct in this section of the Inn River, demonstrated the fishway’s ecological effectiveness^31^.

**Fig. 2:**
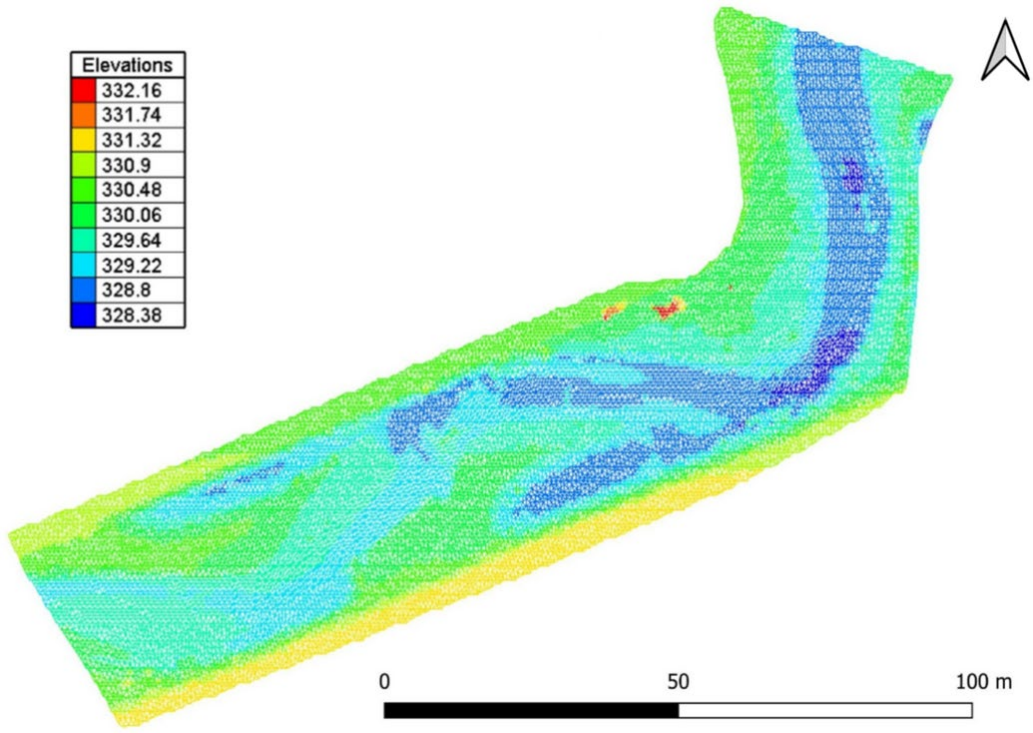
The DEM interpolated onto a numerical mesh. Elevations are in m a.s.l.

This study builds on earlier fieldwork^30,32^, which involved strategically placing two large wood pieces to induce declogging. The data include orthophotos and digital elevation models (DEMs; see Fig. 2) generated through structure-from-motion at a resolution of 0.05 m, as well as sediment samples collected before and after an artificial flood designed to produce morphological changes. Additional topographic data in permanently wetted pools were obtained with a Leica DGPS device with an accuracy of ±0.02 m. The riverbed characteristics quantified in the field were fine sediment fraction (FSF) smaller than 2 mm, full grain size distributions, porosity, hydraulic conductivity, and interstitial oxygen levels. Measurements of flow velocity and water depth, taken with a FlowTracker2 acoustic Doppler velocimetry (ADV) device, were also available from post-flood baseflow conditions.

From the FlowTracker2 measurements, a proxy for estimating the turbulent kinetic energy (TKE) downstream of the large wood logs was derived. This proxy was necessary because the sampling frequency of the FlowTracker2 device did not capture velocity fluctuations at Kolmogorov^33^ micro-scales, which are important for quantifying TKE. The TKE proxy was then normalized by the cross section-averaged flow velocity (*U*) as follows:

1 $$\begin{aligned} {\text {TKE}}_{\text {proxy}} = \frac{1}{2} \left( \overline{\text {u}_{\text {x}}'^2} + \overline{\text {u}_{\text {y}}'^2} + \overline{\text {u}_{\text {z}}'^2} \right) \cdot U^{-2} \end{aligned}$$

where $\overline{\text {u}_{\text {x}}'^2}$, $\overline{\text {u}_{\text {y}}'^2}$, and $\overline{\text {u}_{\text {z}}'^2}$ represent the turbulent velocity fluctuations in the streamwise, transverse, and vertical directions, respectively. The cross-section averaged velocity *U* was obtained from acoustic Doppler current profiler (ADCP) measurements.

### Numerical modeling workflow

A 2d numerical model was constructed using the TELEMAC software suite^29^, which iteratively approximates solutions to the shallow-water equations^34^. Sediment transport and topographic change were indirectly coupled through the Gaia module in TELEMAC^35^. Thus, for each timestep, the shallow-water and sediment transport equations, including topographic change according to the Exner equation^36^, were solved only once, without iterative feedback.

The computational domain was divided into a refined riverbed mesh generated using a channel generator implemented in the BlueKenue software^37^. with a longitudinal spacing of 0.75 m for flow-aligned equilateral elements, and a floodplain mesh composed of scalene triangles with an edge growth ratio of 1.2 and an edge length of 1 m. This approach resulted in a final mesh of 28,167 elements and 14,403 nodes, with an average interior edge length of 0.8 m. The final mesh is shown in Fig. 2. More details on the mesh generation can be found in Supplementary Information SI1.

Eleven friction zones were defined across the computational domain and calibrated hydrodynamically using the available field data. The calibrated model was then employed to evaluate whether the hydro-morphodynamic 2d approach can reliably predict the evolution of FSF in the surface and subsurface layers of a gravel-dominated riverbed.

Fine sediment infiltration modeling in Gaia ^35^ includes vertical mass exchange and stratification within bed layers by dynamically tracking multiple sediment size fractions. The governing equations extend sediment transport formulas (suspended and bedload) by incorporating sediment sorting and interlayer mass exchange, governed by vertical mass balance constraints and empirical relationships. These processes are modeled numerically by solving the vertical layer interaction equations concurrently with the horizontal sediment transport equations, allowing accurate representation of fine sediment infiltration into coarse bed matrices. Detailed descriptions of relevant equations can be found in the SI2.

#### Roughness zones

The channel corridor encompassed multiple morphological units, variations in sediment size, large wood elements, and vegetation patterns. This heterogeneity required the definition of multiple friction zones, each specified by distinct roughness characteristics. For instance, calm backwater zones (swales) contained deposits of fine sediment, while the main channel was predominantly composed of coarse gravel. The grain size compositions adopted for the eleven friction zones are listed in Table 1. In addition, the riverbed was vertically divided into a 0.08 m-thick surface layer and a 0.6 m-thick subsurface layer, allocated according to the dominant roughness heights of each friction zone. These zones also incorporated two large wood logs and their upstream and downstream (wake) regions, recognized as key sites for (de-)clogging processes^30^ (SI3, Fig. S2).

The zonal roughness coefficients were defined following the concept of equivalent friction length^38^ and served as calibration parameters. In gravel-dominated zones, calibration values ranged from 1 to 3 $\times D_{50}$. In sand- and finer sediment-dominated zones, where sediment size was below 1 mm, the roughness length was approximated as 0.5 $\times$ the dune height, estimated at 0.02 m^39,40^. The roughness-height approach accounted for diminished friction effects with increasing water depth. For vegetated zones, the vegetation friction formula from Baptist et al.^41^ was applied. This approach has limitations in that it may lead to an overestimation of vegetation-induced friction in fast currents, but these limitations were acceptable in this study because vegetation was present only in slow-flowing floodplain areas and with limited growth. Further details and discussion of the roughness zones and models used can be found in SI3.

At the inlet and outlet boundaries (i.e., lateral walls), a fixed Manning’s *n* = 0.025 m^-1/3^ s was determined, based on inverse calculations using the Manning-Strickler formula^42,43^ and ADCP measurements (see SI3).

**Table 1:** Grain-size fractions for the 0.08 m-thick surface and 0.6 m-thick subsurface layers, used to define friction zones at model initialization.

Zone name	Riverbed	Floodplains	Boulders	Root wads	Backwater	$\text {Vegetation}^{\dagger }$	$\text {FP}^{*}$	Log A (US)	Log A (DS)	Log B (US)	Log B (DS)
Roughness height (m)	0.01–0.09	0.01–0.09	0.5–2	0.09–0.3	0.09–0.3	0.01–0.09	0.01–0.09	0.01–0.09	0.01–0.09	0.01–0.09	0.01–0.09
Grain size	Fraction contributing to surface layer zones										
$D=0.4 \times 10^{-3}$ m (FSF)	0.1	0	0	0.15	1	0.05	0.5	0.298	0.345	0.137	0.166
$D=7 \times 10^{-3}$ m	0.25	0.25	0	0.7	0	0.25	0.4	0.15	0.08	0.213	0.184
$D=22 \times 10^{-3}$ m	0.5	0.5	0	0.15	0	0.5	0.1	0.5	0.5	0.5	0.5
$D=57 \times 10^{-3}$ m	0.15	0.25	0	0	0	0.2	0	0.052	0.075	0.15	0.15
$D=500 \times 10^{-3}$ m	0	0	1	0	0	0	0	0	0	0	0
Grain size	Fraction contributing to subsurface layer zones										
$D=0.4 \times 10^{-3}$ m (FSF)	0.1	0	0.05	0.15	0.2	0	0.5	0.298	0.345	0.137	0.166
$D=7 \times 10^{-3}$ m	0.25	0.25	0.1	0.7	0.4	0.25	0.4	0.15	0.08	0.213	0.184
$D=22 \times 10^{-3}$ m	0.5	0.5	0.05	0.15	0.4	0.5	0.1	0.5	0.5	0.5	0.5
$D=57 \times 10^{-3}$ m	0.15	0.25	0	0	0	0.25	0	0.052	0.075	0.15	0.15
$D=500 \times 10^{-3}$ m	0	0	0.8	0	0	0	0	0	0	0	0

^†^Vegetation roughness coefficient was calculated according to Baptist et al.^41^ using a bulk drag coefficient of 1.0 (rigid cylinder), vegetation density (=1/spacing^2^) with spacing of 0.1 m, vegetation diameter of 0.055 m, and length of 0.4 m.

*FP fine particle zones.

#### Initial conditions, boundaries, and simulation procedure

**Fig. 3:**
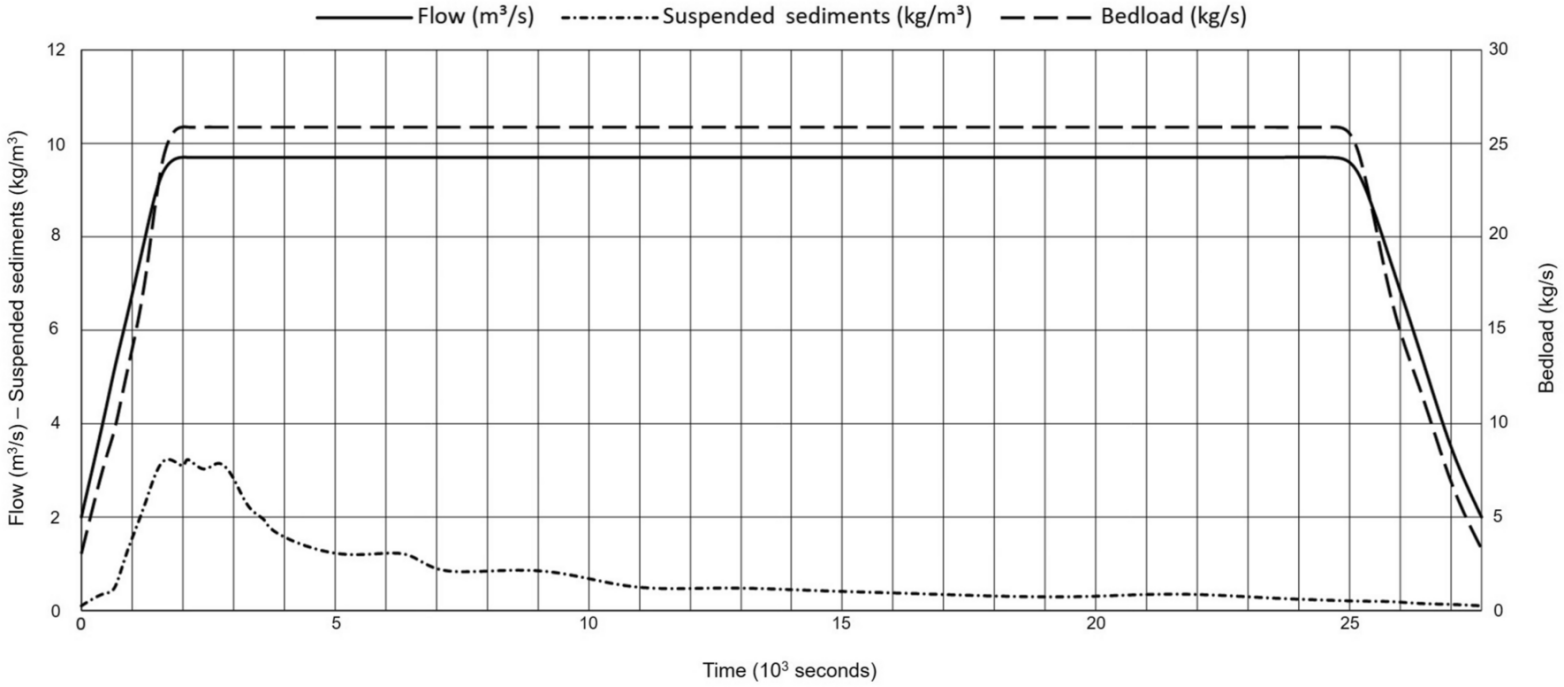
The hydrograph of the artificial flood in $\hbox {m}^3$ $\hbox {s}^{-1}$ with suspended load in $\hbox {kg}~\hbox {m}^{-3}$, and bedload in $\hbox {kg}~\hbox {s}^{-1}$ influx.

Initially, steady baseflow conditions ($2~\hbox {m}^3$ $\hbox {s}^{-1}$) were simulated to calibrate the roughness coefficients of boundary walls. The simulation continued until inflow and outflow stabilized. The resulting steady-state hydraulic outputs (e.g., flow velocity and water depth at each mesh node) then served as initial conditions for quasi-steady simulations of the artificial, morphologically effective flood hydrograph (Fig. 3). In the field, the hydrograph was imposed by operating a weir at the fishway intake. In the numerical model, the hydrograph was prescribed as a discrete, time-variate inflow boundary condition. The outflow boundary condition was defined by a rating curve, namely a stage-discharge relationship at the model exit, based on measured and interpolated discharge-depth pairs at baseflow and peak flow.

Sediment transport boundaries were specified as suspended load concentrations in $\hbox {kg}~\hbox {m}^{-3}$ and bedload transport rates in $\hbox {kg}~\hbox {s}^{-1}$. The suspended load concentrations were derived from field measurements. Bedload influxes were calculated using the Wong-Parker correction^44^ of the Meyer-Peter and Müller formula^45^, which is considered valid for this controlled alluvial channel. Both the suspended load and bedload influx (solid discharge) are shown in Fig. 3. At the model outlet, the bed elevation was fixed to ensure numerical stability and prevent unrealistic sediment accumulation or erosion that could arise from unconstrained sediment transport fluxes. This approach was adopted to maintain a stable sediment flux balance and avoid unintended boundary effects that could influence the results within the computational domain. Other sediment transport parameters were left unconstrained.

The hydrodynamic calibration involved extracting the simulated flow velocity and water depth from the final simulation timestep at the measurement locations. The model accuracy was assessed by comparing these post-flood flow velocities and water depths with the measurements. The comparison was done using the root mean square error (RMSE), which quantifies the difference between the observed and predicted values. Specifically, the RMSE was calculated as:

2 $$\begin{aligned} {\text {RMSE}} = \sqrt{\frac{1}{n} \sum _{i=1}^{n} \left( y_i - \hat{y}_i \right) ^2} \end{aligned}$$

where $y_i$ is the measured value, $\hat{y}_i$ is the simulated value, and *n* is the number of data points. Small RMSE indicates high accuracy, that is, low error between the observed and predicted values. The RMSE calculation guided the optimization of the roughness heights (see Table 1).

Additionally, the Shields parameter^46^ was refined to align simulated topographic changes with those observed during the artificial flood. Its initial value of 0.047 was adjusted by up to 50%. This parameter also influenced the suspended load concentration, modeled through the Van Rijn formula^47^.

The calibrated model then served to test whether the 2d hydro-morphodynamic model could identify clogging-prone zones based on changes in FSF within the substrate layers. Specifically, measured FSF was compared to area-averaged simulated FSF in the surface and sublayers after the artificial flood.

### Detection of clogging

The simulation capacities for detecting riverbed clogging were tested in the vicinity of the two large wood logs (log A, located upstream of log B). These logs were placed to induce declogging, which was partially verified based on FSF, porosity, and dissolved oxygen measurements in the field^30,32^. Because the numerical model used here could not technically evaluate porosity and oxygen concentrations, only changes in FSF were examined to assess clogging risk.

Field analyses showed that log A efficiently generated hydro-morphodynamic changes during the artificial flood, reducing FSF upstream and in its wake. Log B was less efficient at declogging, and even led to small FSF increases upstream and in its wake^30^. These field values, obtained using MultiPAC^17^, indicate a declogging effect around log A that was not observed for log B. Specifically, eight MultiPAC measurements (two sets at four locations) were taken upstream and downstream of each log before and after the artificial flood event. These measurements provided grain size distributions, including FSF, as well as porosity and vertically resolved oxygen and hydraulic conductivity profiles.

To be viewed as a reliable predictor of riverbed clogging, the numerical model had to reproduce at least the FSF trends of decreasing values near log A and increasing values near log B after the artificial flood. It also needed to reflect topographic changes since newly deposited gravel and erosion zones can disrupt or remove clogging. Finally, suspended load measurements aided in evaluating the capacity of the model to simulate fine (suspended) sediment transport.

## Results

### Calibration parameter optima

The iterative model calibration regarding simulated and observed flow velocity and water depth after the artificial flood yielded the roughness heights listed in Table 2. These optima were found within the parameter ranges given in Table 1, based on the flow velocity and water depth RMSE in the vicinity of the logs (see the RMSE results in Table 4).

**Table 2:** Calibrated roughness heights for the roughness zones introduced in Table 1.

Zone	River-bed	Flood-plains	Boulders	Root wads	Back-water	Vege-tation*	FP	Log A (US)	Log A (DS)	Log B (US)	Log B (DS)
$\text {k}_{\text {s}}$ (m)	0.066	0.075	1.5	0.22	0.01	0.07	0.01	0.066	0.066	0.066	0.066

*For vegetation friction; drag coefficient = 1, density $\times$ diameter = 5.5, Vegetation length = 0.4.

Similarly, the Shields parameter was calibrated for the grain diameter classes (see rows in Table 1). The detailed calibration procedure is described in SI4, and the optimum Shields parameter values are summarized in Table 3.

**Table 3:** Calibrated values of the Shields parameter for relevant grain diameter classes defined in Table 1.

Grain diameter (m)	$0.4\times 10^{-3}$ (FSF)	$7\times 10^{-3}$	$22\times 10^{-3}$	$57\times 10^{-3}$	$22\times 10^{-3}$
Shields parameter (–)	0.047	0.053	0.054	0.055	0.055

The water depth and flow velocity RMSEs (Eq. 2) were calculated in the near-log regions where measurements were available, that is, patches of particular interest for analyzing clogging. The plots showing the measured and modeled water depth and flow velocity results are provided in SI4. The RMSEs indicate that the water depth and flow velocity near log B were less accurately simulated than those near log A (Table 4).

**Table 4:** RMSE between the simulated and measured flow velocities.

Location	Water depth RMSE (m)	Velocity RMSE ($\hbox {m}~\hbox {s}^{-1}$)
Vicinity of log A	0.083	0.2
Vicinity of log B	0.30	0.3

**Fig. 4:**
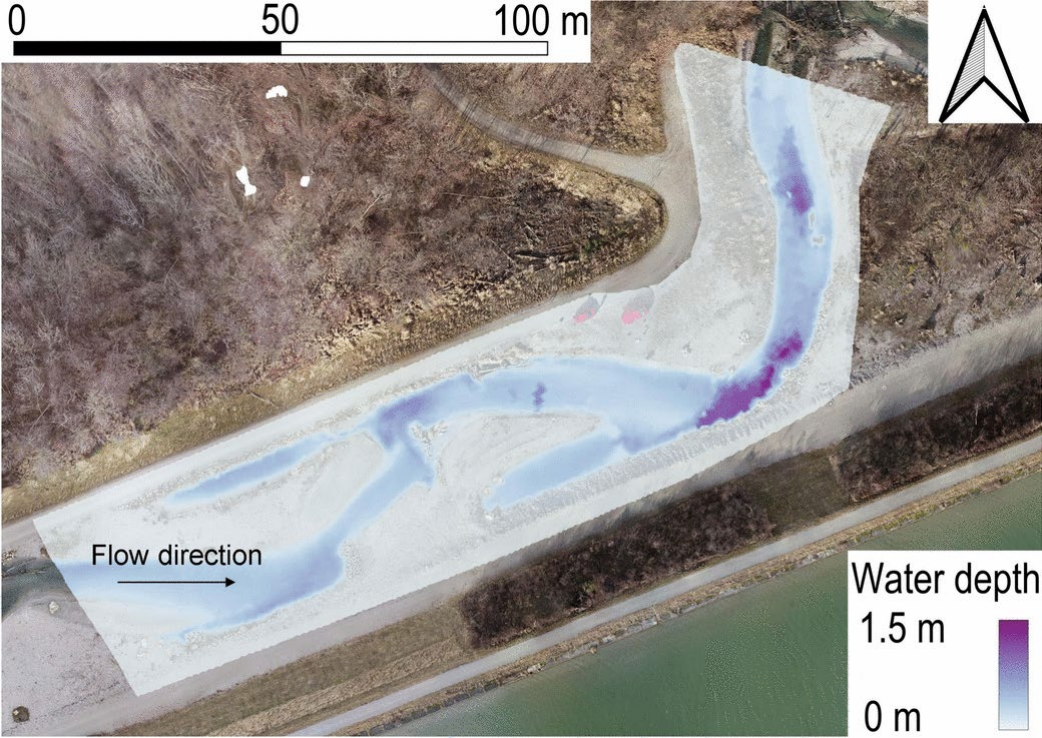
Qualitative overlay of the water depth and the observed wetted boundary of the fishway at baseflow before the flood event. The fishway is visible on drone imagery in the background.

In addition to the post-flood RMSE comparison (Table 4), a qualitative assessment of the pre-flood water depths, overlaid with drone imagery captured before the artificial flood event, is presented in Fig. 4. This plot indicates strong agreement between numerically simulated and observed water depths, suggesting that the model accurately simulates the wetted boundaries.

### Hydrodynamics

**Fig. 5:**
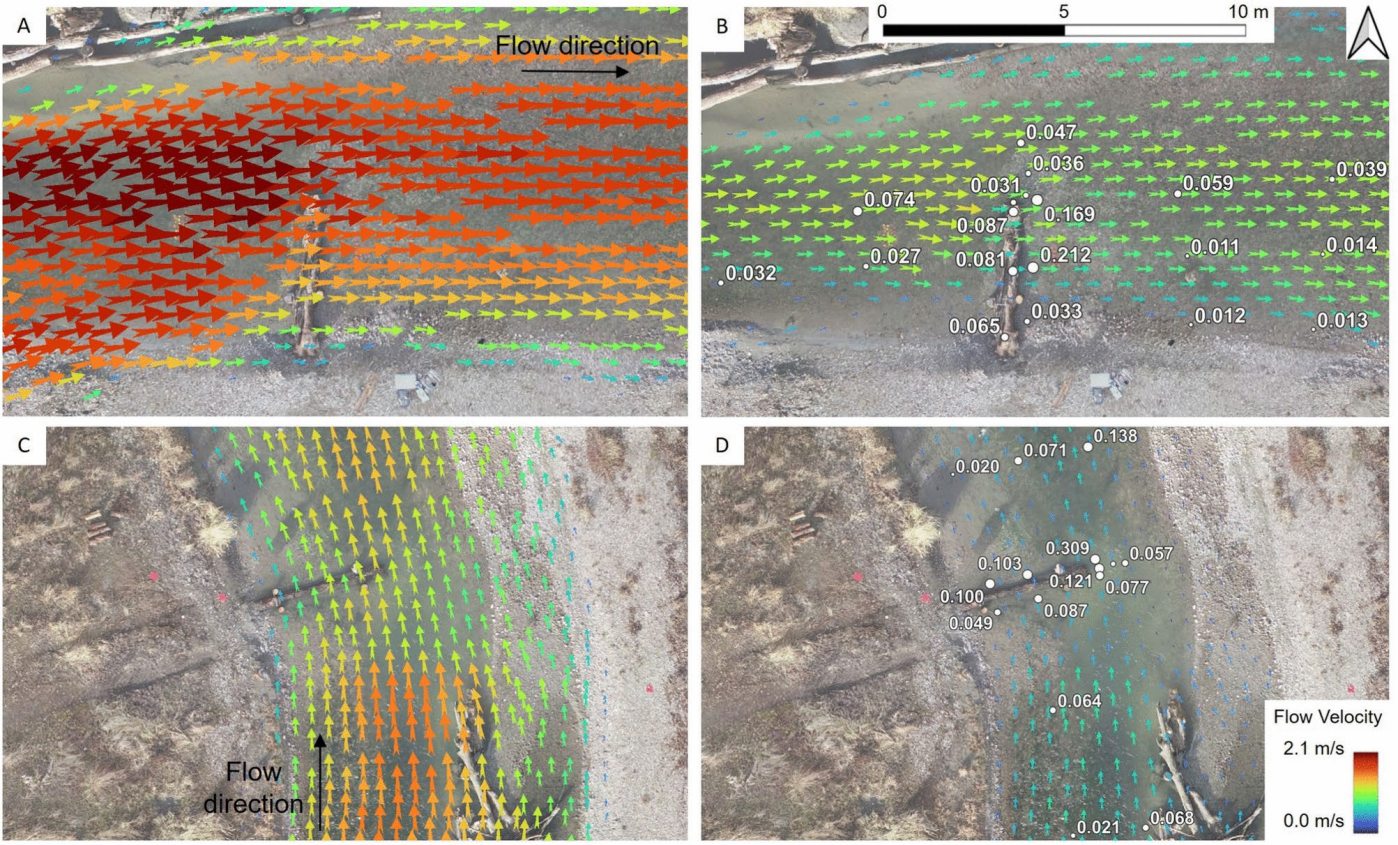
Velocity vectors in the vicinity of log A (graphs **A** and **B**) and log B (graphs **C** and **D**), at the end of the peak flow period of 9.7 $\hbox {m}^3$ $\hbox {s}^{-1}$ (graphs **A** and **C**) and after the artificial flood at baseflow conditions of 2 $\hbox {m}^3$ $\hbox {s}^{-1}$ (graphs **B** and **D**). White points show the TKE proxy measured at baseflow conditions.

Patches with low flow velocity during peak discharge can be linked to an increased tendency for (fine) sediment deposition, indicating patches at risk of surface riverbed clogging. Figure 5 presents the velocity vectors around log A and log B, alongside the TKE proxy (Eq. 1) derived from FlowTracker2 measurements at baseflow conditions, after the flood event. The magnitudes and directions of the vectors (arrows) represent the horizontal (2d) flow velocity. Log A was located in a faster-flowing and shallower patch than log B, suggesting a higher risk of fine particle deposition in the vicinity of log B, especially in its wake at baseflow conditions.

After the artificial flood, the measured TKE proxy was particularly high immediately downstream of the logs, corresponding to patches where the logs acted similarly to a submerged groin. These elevated TKE values can be associated with scour pools in which turbulent eddies keep fine sediment in suspension, thus reducing the risk of surface clogging. However, this also implies that water with high suspended load concentration could infiltrate the loose gravel deposits downstream of the logs, indicating a higher risk of internal clogging.

### Topographic change

**Fig. 6:**
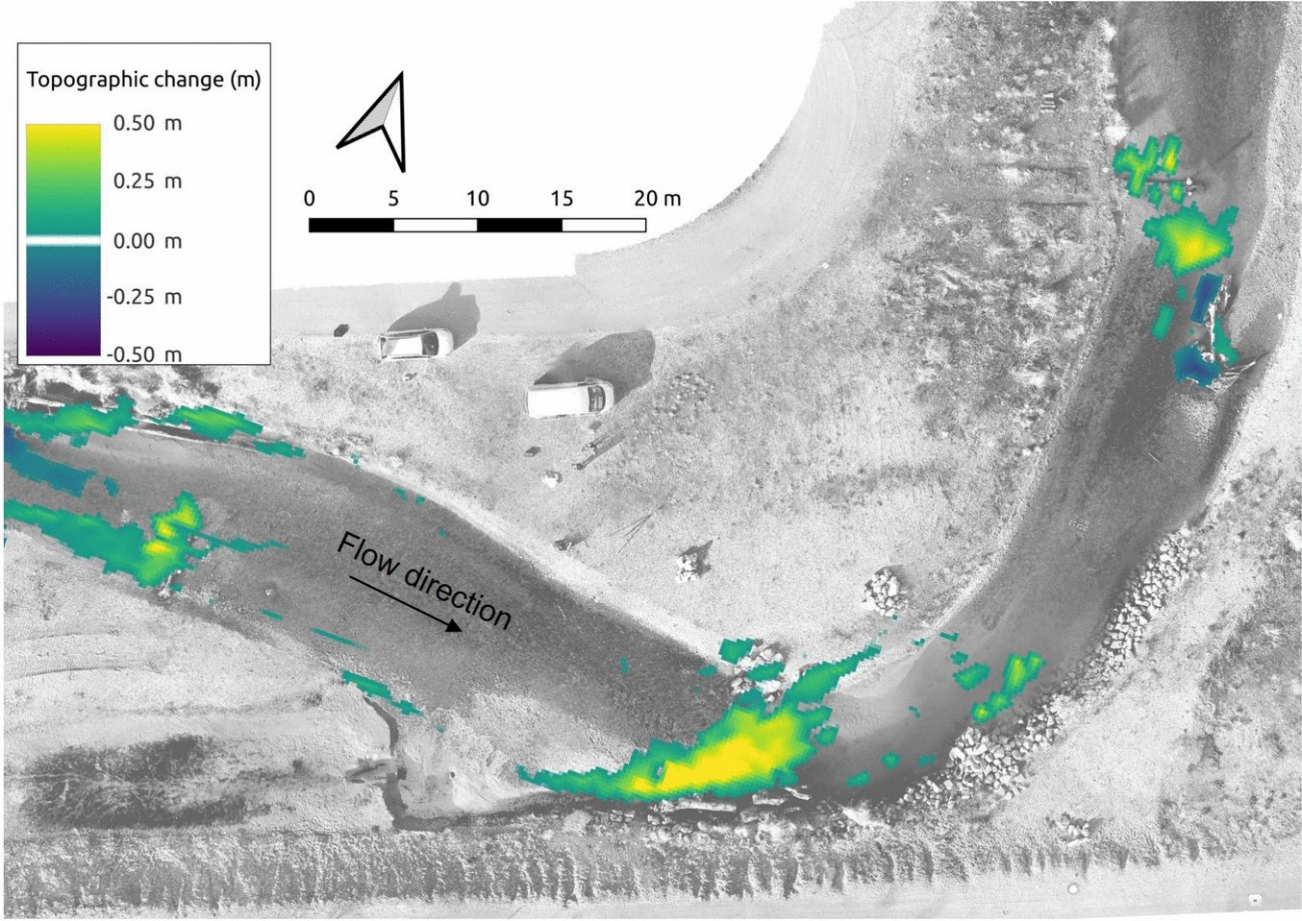
DEM of Differences (DoD) illustrating simulated topographic changes. Deposition is represented by positive values (yellow to green tones). Erosion corresponds to negative values (blue to purple tones).

Topographic change maps reveal distinct zones of erosion and deposition, with pronounced erosion-deposition patterns indicating high morphodynamic activity that likely prevents riverbed clogging due to the washing out of fine sediment. To quantify topographic change, pre- and post-flood elevations were subtracted on a pixel-by-pixel basis, excluding any differences within the measurement accuracy of ±0.1 m, thereby generating a DEM of Differences (DoD). The complete workflow is described in SI5. The resulting DoD (Fig. 6) highlights deposition (yellow to green tones) and erosion (blue to purple tones) patches. Deposition peaked at 0.67 m in the bottom-right corner. Erosion reached − 0.28 m at the right bank upstream of log B. These simulated patches match DGPS measurements within ±0.1 m across the study reach, indicating that the model accurately simulated observed topographic changes. Downstream of log A, small deposition patches in the DoD align with field observations, whereas larger deposition occurred upstream of log A. Deposition was more pronounced in the wake of log B, presumably due to the decelerating effect of the log during the flood. The substantial deposit between logs A and B (center-bottom of Fig. 6) marks a riffle that migrated downstream, affirmed by DGPS data and qualitative field observations.

### Fine sediment fraction (FSF) of the riverbed

The measured and numerically simulated FSFs at the four MultiPAC measurement points upstream and downstream of the two logs are listed in Table 5. Field measurements represent average FSFs within the upper 0.6 m to 0.7 m of the riverbed, while the numerical model domain considered a depth of 0.68 m, comprising the 0.08 m surface layer and a 0.6 m subsurface layer. Post-flood field measurements showed a reduction in FSF upstream and downstream of log A, indicating diminished physical clogging in this zone. In contrast, an increase in FSF was observed in the vicinity of log B, implying enhanced clogging there. The numerical model reproduced the general patterns seen in the field, showing a decrease in FSF near log A and an increase near log B, although discrepancies between measured and simulated FSF magnitudes are evident.

**Table 5:** Measured and simulated fine sediment fractions (FSFs) of the surface (top 0.08 m) and subsurface (0.08–0.68 m) layers from before ($t_0$) and after ($t_1$) the artificial flood event.  The average values of the numerical model at $t_0$ corresponded to the measured averages at $t_0$.

Location	Measured FSF $t_0$ (–)	Measured FSF $t_1$ (–)	Modeled FSF $t_1$ (–) (layer average)	Modeled FSF $t_1$ (–) (surface layer)	Modeled FSF $t_1$ (–) (subsurface layer)
log A US	29.8	22	19.5	1	22
log A DS	34.5	30.2	20.4	1	23
log B US	13.7	13.8	21.05	29	20
log B DS	16.6	18.1	31.5	50	29

**Fig. 7:**
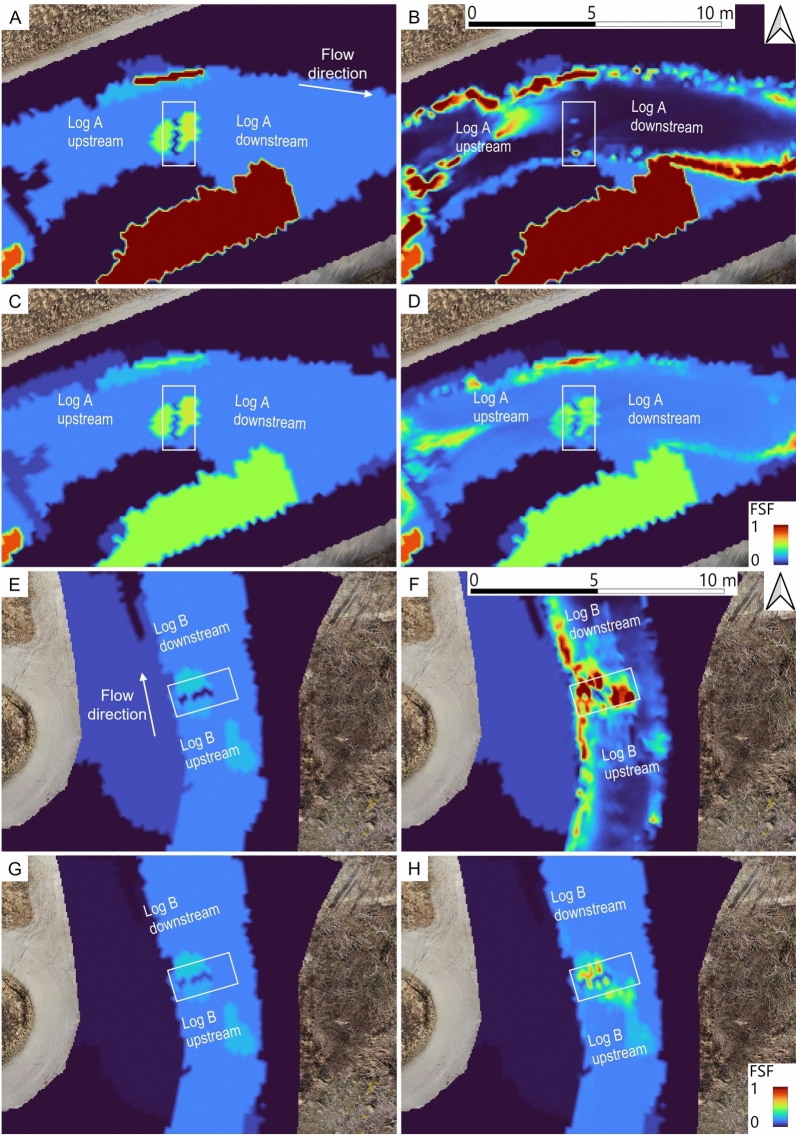
FSF of the surface layer at log A (graphs **A** and **B**; 0.08 m thick) and at log B (graphs **E** and **F**; 0.08 m thick) and the subsurface layer of the riverbed at log A (graphs **C** and **D**; 0.6 m thick) and at log B (**G** and **H**; 0.6 m thick). The FSF at the initial state is shown in graphs (**A**), (**C**), (**E**) and (**G**). Graphs (**B**) and (**F**) show the FSF of the surface layer. Graphs (**D**) and (**H**) show the FSF of the subsurface layer at the end of the simulations.

The numerical simulations offer a broader view of the FSF patterns around the two logs than is possible from four distinct (MultiPAC) measurement points. The FSFs before ($\hbox {FSF}_{t_0}$) and after ($\hbox {FSF}_{t_1}$) the artificial flood, within the 0.08 m-thick surface layer and the 0.6 m-thick sublayer, are illustrated in Fig. 7. A comparison of Fig. 7 A,B shows that at log A, the initially high FSF values exceeding 0.5 in the surface layer were nearly removed by the artificial flood event. The subsurface layer around log A also shows a decrease in FSF, though it is less pronounced (Fig. 7 C,D). In contrast, a comparison of Fig. 7 E,F reveals a substantial increase in FSF in the surface layer at log B following the flood. The upward trend in FSF near log B is likewise evident in the subsurface layer (Fig. 7 G,H).

### Suspended sediment concentration

**Fig. 8:**
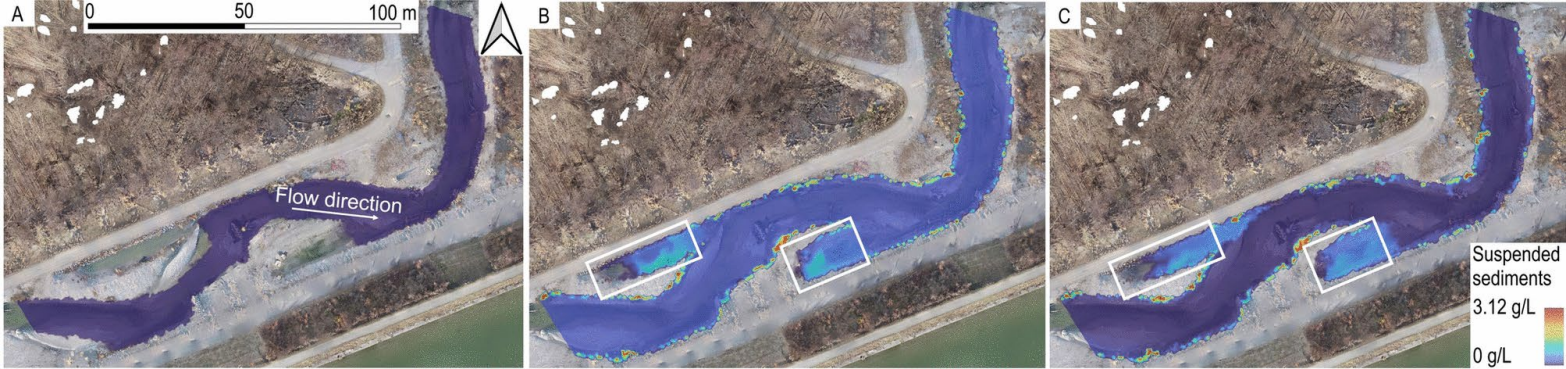
The suspended sediment concentration (SSC) in the water column (**A**) before, (**B**) during (halftime), and (**C**) at the end the artificial flood. Graphs (**A**) and (**B**) correspond to a peak discharge of 9.7 $\hbox {m}^3$ $\hbox {s}^{-1}$. Graph (**C**) corresponds to baseflow conditions of 2 $\hbox {m}^3$ $\hbox {s}^{-1}$.

The suspended sediment concentration (SSC) maps in Fig. 8 show the depth-averaged concentrations of suspended particles finer than 0.5 $\times$ $10^{-3}$ m in the water column. These maps depict conditions at the onset, halfway through, and end of the artificial flood event. The time-dependent SSCs at the upstream boundary were prescribed based on the available measurements (see Fig. 3). SSC remained low in the channel center, indicating effective transport of suspended particles and thus, a reduced likelihood of clogging in the main flow path. Higher SSCs were observed in the calmer water patches (swales) at the left and right banks in the center of the images. However, elevated SSC in the water column does not necessarily imply that fine sediment was deposited. Still, the swale that maintained high SSC toward the end of the flood event (Fig. 8C) was also found to be covered with a thick mud layer in the fishway prototype. Also, high SSC patches at the right bank, downstream of log A (visible in Fig. 8 B,C), likely contributed to the high FSF patches observed further downstream along the right bank (see Fig. 7 A).

## Discussion

The validation of the numerical model was based on spatially explicit measurements collected on-site, including water depth, flow velocity, bed elevation, and FSF. However, the use of 2d simplifications led to some discrepancies between observed and simulated results, particularly in the vicinity of log B, where the RMSEs for water depth and flow velocity were higher compared to log A (see Table 4). These differences can be related to the contrasting environments of the logs: log A was located in a shallower, generally faster-flowing area, while log B was situated in a deeper area with lower flow velocities (see Fig. 5). In the deeper region around log B, 3d flow processes exert a stronger influence and were less effectively captured by the 2d model. Specifically, circular bend flow and flow separation at the log tip were not fully reproduced, which helps explain the small differences between measured and simulated values. While the 2d model captures many primary flow dynamics, it faces challenges in fully reproducing more complex 3d flow processes in deeper zones, such as around log B. These 3d effects, including secondary currents and vertical stratification, can strongly influence near-bed sediment transport and deposition patterns. Secondary currents occur primarily in bends, where spiral flows drive finer sediment toward the inner bend near the bed and cause erosion on the banks of the outer bend. The logs considered here were not in bends, although there was a sharp left bend between the two logs, with fine sediment deposits on the bank of the inner bend. The outer bend was protected with boulders to prevent erosion. Vertical stratification can also cause flow velocity variations across the water column, affecting sediment transport, especially for finer particles in deep flows. Since deeper flows up to approximately 1 m water depth occurred only in the vicinity of log B, the 2d simplification hypothesis may explain why some of the observed sediment distribution and FSF trends around log B were not fully replicated in the 2d simulations.

The two sets of four MultiPAC measurements showed reductions in FSF near log A, with decreases of 7.8% upstream and 4.36% downstream, suggesting de-clogging in this area, which aligns with regions of high horizontal flow velocity at the flood peak (cf., Fig. 5A). In contrast, the patch around log B exhibited FSF increases of 0.14% upstream and 1.53% downstream. Flow velocity magnitudes near log B were generally lower (cf., Fig. 5C), indicating that less sediment was mobilized and more sediment was deposited around log B. Although the numerical model reproduced the measured FSF trends, some discrepancies arose regarding the magnitude of FSFs, particularly around log B (Table 5). Smaller changes observed in the field data, compared to the model, may stem partly from limitations in simulating flow velocity and water depth near log B. Furthermore, while the field data did not distinguish between surface and subsurface layers, the numerical model specifies these layers.

**Fig. 9:**
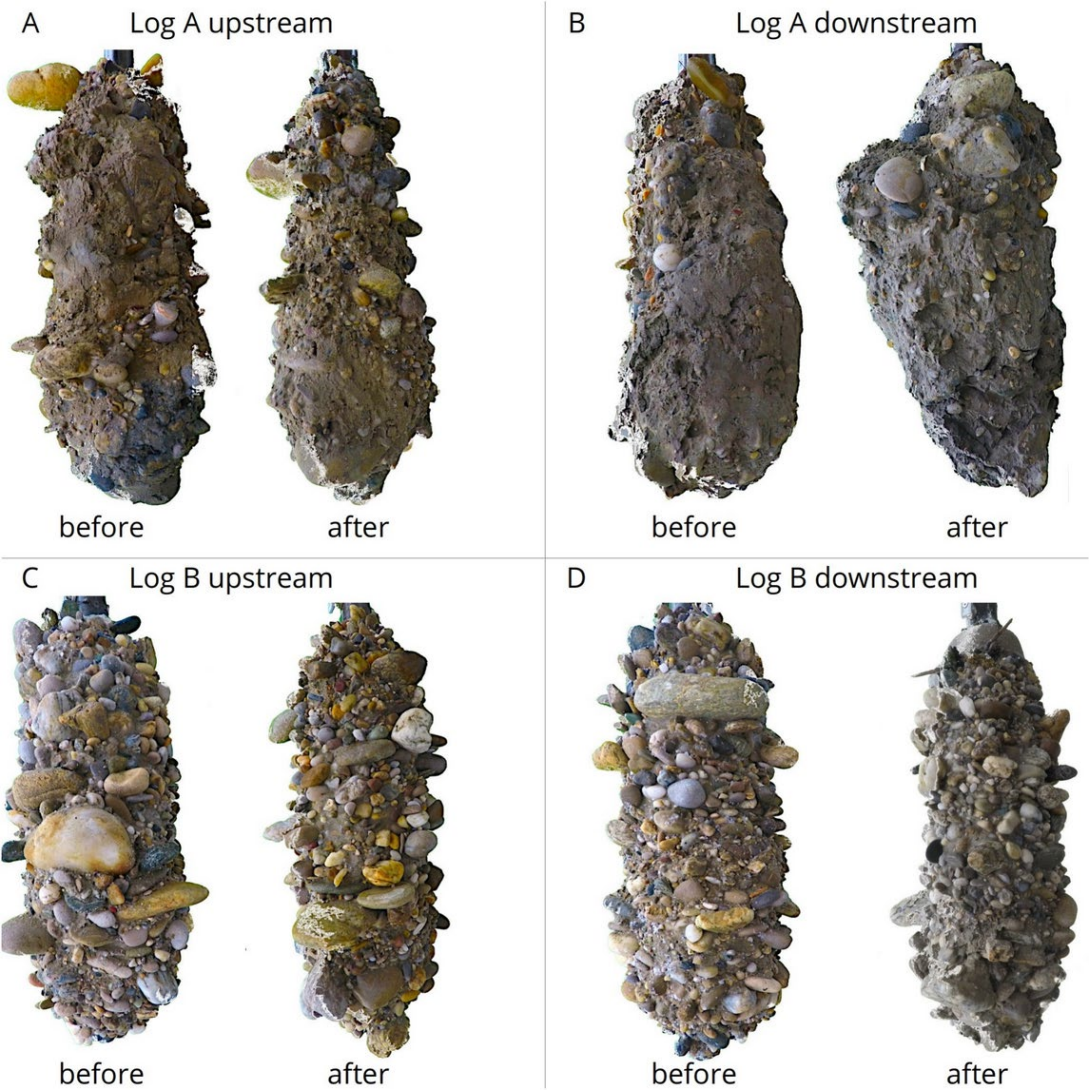
Frozen cores extracted upstream and downstream of log A and log B in the framework of MultiPAC measurements, before and after the artificial flood. Each frozen core was approximately 0.6–0.7 m long.

The simulated topographic changes showed slightly more pronounced deposition upstream of log B than around log A (cf., Fig. 6), yet these deposits were much finer near log B (cf., Fig. 7). Pictures of the frozen sediment cores (Fig. 9) provide a better understanding of the observed and simulated refinement trends, especially regarding the differentiation between surface and subsurface layers downstream of log B. A comparison of the pre- and post-flood cores downstream of log A (Fig. 9B) shows coarser gravel grains confined to the upper surface layer. Similarly, downstream of log B (Fig. 9D), a fine sediment deposit is evident at the surface (top) of the “after” core, consistent with the numerically predicted refinement in the surface layer. This fine sediment deposition explains the simulated increase in FSF in the surface layer and shows that the high simulated FSF refinements are eventually also supported by field observations.

The simulated SSC patterns provided insight into fine particle transport dynamics. While high SSC in the water column (Fig. 8) did not generally correspond to FSF patterns (Fig. 7), one notable high SSC patch emerged along the right bank downstream of log A, toward the end of the flood simulations (center of Fig. 8B,C). In addition, downstream of these elevated SSC spots, water with high SSC fed by the swale (backwater) on the right bank floodplain downstream of log A contributed to the development of a high FSF patch along the right bank downstream of the swale (Fig. B). These high FSF patches suggest “fine sediment pollution” caused by fine-particle export from the swale, which is well indicated by the numerical model. This observation illustrates how fine sediment deposits on the bank or floodplain can contribute to clogging and how a numerical model can help pinpoint the triggers of clogging hotspots.

Clogging is associated not only with high FSF but also with steep oxygen reductions in the riverbed along the vertical axis^17,30^, diminished hydraulic conductivity^19,24^, and reduced sediment matrix porosity^23^. However, to the best of the Authors’ knowledge, there are currently no simulation tools capable of predicting how fine sediment deposition alters oxygen concentration or hydraulic conductivity. Changes in porosity can be inferred from the grain size distribution^19^, and therefore, porosity can be considered as a surrogate parameter for the outcomes of a predictive 2d numerical model. Although there are existing models that estimate how deep fine particles may penetrate the substrate^48,49^, such approaches are designed for reproducing (i.e., hindcasting) clogging processes with limited predictive power. Also, this study focused specifically on physical clogging, but biological mechanisms such as biofilm colonization and organic matter accumulation also contribute to clogging^4,11,50–52^.

Biofilm growth can enhance physical clogging by reducing interstitial pore space, increasing sediment cohesion, and further decreasing hydraulic conductivity, thereby also exacerbating fine sediment retention^53^. Additionally, microbial activity can influence oxygen availability in the upper layer of the hyporheic zone, as biofilm consumes oxygen and promotes anoxic conditions, which can accelerate sediment consolidation and alter flow paths within the riverbed^54^. While these interactions are well-documented, simulating the interplay of physical and biological processes remains challenging due to the complexity of model coupling and the extended timescales required for biological growth, which can range from weeks to several decades.

Ultimately, concerning the hypothesis that a 2d hydro-morphodynamic model can predict FSF changes in gravel-bed rivers in the framework of physical clogging analysis, this investigation did not yield evidence to the contrary. Despite the limitations inherent in a 2d (rather than 3d) approach, key FSF trends around the two investigated wood logs were effectively reproduced. Therefore, FSF trends for future flood scenarios can be predicted using this numerical model.

## Conclusions

This study evaluated the capacity of a two-dimensional hydro-morphodynamic model to simulate riverbed clogging dynamics during an artificial flood event, focusing on fine sediment transport and deposition near two large wood pieces. The simulation successfully captured fine sediment dynamics, which are primary drivers and indicators of clogging and de-clogging. In particular, the observed reduction in fine sediment fractions upstream and downstream of one of the large wood pieces located in a shallow, fast-flowing area was consistent with field measurements. This finding underscores the ability of the model to represent key hydrodynamic processes behind fine sediment dynamics and (de-)clogging. The model also effectively distinguished fine sediment exchange between surface and subsurface sediment layers critical for differentiating between surface and internal clogging. Limitations emerged when simulating the vicinity of a large wood piece surrounded by deeper, slower-flowing water. These shortcomings appear attributable to the simplifications inherent in computationally efficient two-dimensional simulations. Despite these constraints, the results demonstrate that identifying clogging-prone zones is feasible with such models. Moreover, fine sediment deposits on banks and floodplains were found to cause increased fine sediment infiltration downstream, suggesting that such floodplain deposits exacerbate physical clogging. These outcomes are valuable for ecohydraulic research and highlight the potential of numerical hydro-morphodynamic simulation tools to guide river management and habitat restoration efforts, while also emphasizing the need for improved three-dimensional models to capture processes relevant to riverbed clogging.

## Supplementary Information

Supplementary Information.

## Data Availability

The model and data used and produced by this study are available at https://doi.org/10.5281/zenodo.14765949^55^. Documentation for Telemac can be found on the developer’s website http://www.opentelemac.org. The pre-processing, simulation, and post-processing workflow pipelines used in this manuscript are provided at https://hydro-informatics.com/numerics/telemac/telemac.html.
