# Supplementary material for: Hydro-morphodynamic numerical modeling indicates risk zones for riverbed clogging
Source: Sci Rep. 2025 Mar 29;15:10873. doi: 10.1038/s41598-025-95150-3 (PMC11954975; doi:10.1038/s41598-025-95150-3)
Supplement: Supplementary file 1 — Supplementary Information. [file 41598_2025_95150_MOESM1_ESM.pdf]

Supplementary Information for

Hydro-morphodynamic numerical modeling indicates risk zones for riverbed clogging

(Scientific Reports)

Authors: Federica Scolari, Mohammed Fadul, and Sebastian Schwindt

SI 1 Mesh generation

The model domain comprises two numerically distinct areas: the riverbed and the floodplain. The riverbed represents the permanently wet region at a flow rate of 2 m^3 s^{-1}. For the riverbed, BlueKenue's T3 channel generator was used to define 15 cross-channel nodes with dynamic spacing based on the wetted riverbed boundaries. The longitudinal spacing between nodes was set to 0.75 m, which was determined through an iterative trial-and-error process to be the finest resolution compatible with the chosen coordinate reference system (smaller values resulted in mesh errors due to duplicated coordinates). This process produced a flow-aligned mesh predominantly composed of equilateral triangular elements, reducing lateral numerical diffusion, enhancing computational efficiency, and improving result accuracy[1]. The floodplains were inundated by the peak flow. BlueKenue's T3 mesh generator was used to create scalene triangles with an edge growth ratio of 1.2 and an edge length of 1 m. It was also used to integrate the floodplain mesh with the previously generated riverbed mesh. The final mesh, shown in Figure S1, consisted of 28,167 elements and 14,403 nodes, with a mean interior edge length of 0.8 m.

Although the longitudinal spacing in the riverbed was primarily constrained by coordinate system limitations, additional tests were conducted to assess the suitability of the selected mesh resolution for hydraulic simulations. Coarser resolutions led to reduced numerical stability and a loss of detail in key flow patterns, particularly in regions of higher velocity gradients and recirculation zones. The chosen resolution was therefore confirmed as a balance between computational efficiency and hydraulic accuracy.

In the vertical direction, the computational domain was discretized using a layered approach, with finer resolution (0.08 m) near the water surface and coarser resolution (0.6 m) in the subsurface. This division was chosen to better resolve near-surface flow dynamics and turbulence, where velocity gradients and free-surface interactions are more pronounced. In contrast, deeper regions experience lower velocity gradients, allowing for coarser discretization without significant loss of accuracy.

SI 2 Governing equations for sediment transport and bed changes

The numerical simulations presented in this manuscript were performed with the Gaia module of TELEMAC, which allows indirect coupling of bed change and hydraulics to assess fine sediment infiltration into two substrate layers. The Gaia module calculates sediment transport, bed evolution and mass exchange with the bed using the specific equations. In particular, suspended sediment was modeled according to van Rijn (1984)[3], the Meyer-Peter & Müller (1948)[4] with the Wong-Parker (2006)[5] correction was used for bedload modeling, topographic change was modeled according to Exner (1925)[6], and mass exchange between active and substrate layers was modeled according to Hirano (1971)[7] (see detailed discussions and amendments in Blom (2008)[8] and Stecca *et al.* (2016)[9]).

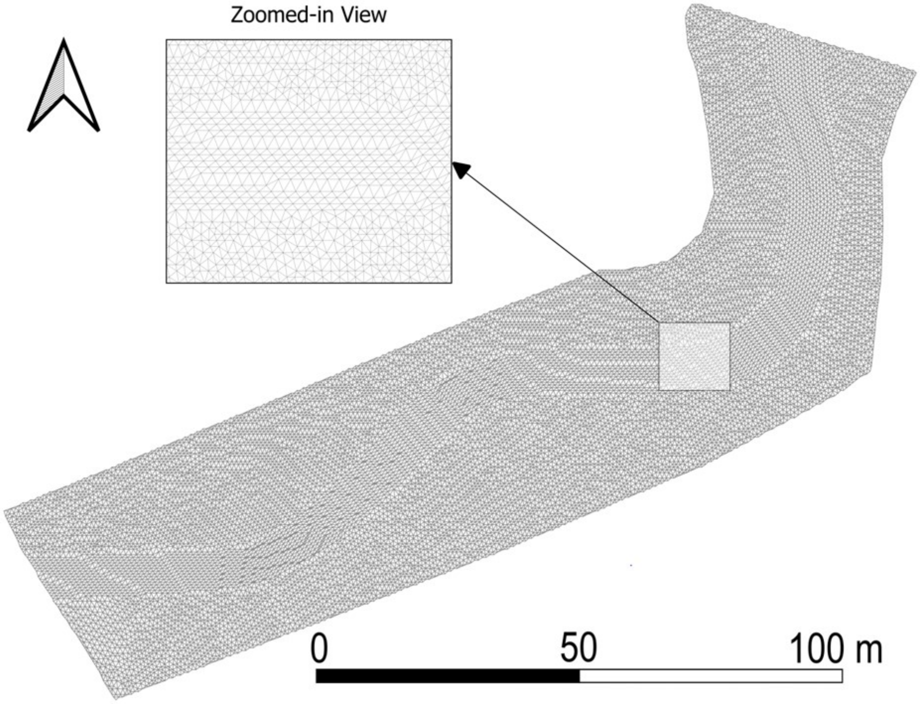

Figure S1. Final mesh with 14,403 nodes and a mean interior edge length of 0.8 m, generated with BlueKenue[2].

Bed evolution & mass exchange

Bed level change due to sediment erosion and deposition processes is governed by the Exner (1925)[6] equation, expressing mass conservation in the sediment bed:

$$(1-\eta_p)\frac{\partial z_b}{\partial t} = -\nabla \cdot \mathbf{q_b} + (D-E) \tag{1}$$

where z_b is the bed elevation; η_p is the dimensionless sediment porosity; $\mathbf{q_b}$ is the bedload transport vector; and E and D refer to erosion and deposition fluxes for suspended sediment. Specifically, erosion E and deposition D fluxes quantify sediment exchange between the water column and the bed surface:

$$E = \omega_s C_{eq} \tag{2}$$

$$D = \omega_s C_s \tag{3}$$

where ω_s is the sediment settling velocity (m s^{-1}), calculated based on sediment particle characteristics; C_{eq} is the equilibrium suspended sediment concentration at the bed estimated according to the equilibrium sediment transport relation presented in van Rijn (1984)[3]; and C_s is the depth-averaged suspended sediment concentration (referred to as SSC in the main manuscript). Thus, suspended sediment concentration, E, and D were modeled according to the detailed description in van Rijn (1984)[3] and the Gaia user documentation (see also Tassi *et al.* (2023)[10]).

Suspended load modeling

The suspended load is represented by the advection-diffusion equation for suspended sediment concentration:

$$\frac{\partial (hC_s)}{\partial t} + \nabla \cdot (h\mathbf{U}C_s) = \nabla \cdot (h\Gamma_s \nabla C_s) + E - D \tag{4}$$

where h is the water depth; $\mathbf{U}$ is the depth-averaged flow velocity vector; Γ_s is the sediment diffusivity coefficient tensor

(typically related to turbulent diffusivity); and E and D represent the above-introduced erosion (entrainment) and deposition fluxes, respectively.

Bedload transport

Bedload transport $\mathbf{q_b}$ in this study was computed using the Meyer-Peter-Müller (1948)[4] (in the following referred to as MPM) formula with the correction by Wong and Parker (2006)[5]. In its original form, the dimensionless transport rate q_b^* is expressed as a function of the dimensionless shear stress τ_*:

$$q_b^* = 8\,(\tau_* - \tau_{*c})^{1.5}\,, \tag{5}$$

where τ_{*c} is the critical Shields stress[11]. Dimensionalizing q_b^* uses:

$$q_b = q_b^* \cdot (s-1) \cdot D \cdot \sqrt{g \cdot D} \cdot \rho_s^{-1} \tag{6}$$

with g the gravitational acceleration, D the characteristic grain diameter, and $s = \rho_s/\rho$ the relative density of the sediment. Wong and Parker (2006)[5] revised the coefficients to better align with a broader set of flume data, providing updated exponents and critical stress values:

$$q_b^* = 4.93\,(\tau_* - 0.0495)^{1.6} \tag{7}$$

This correction was relevant in this study because its purpose is to improve predictions over a wider range of grain sizes, gradients, and flow conditions while maintaining the original form of the MPM formula.

SI 3 Roughness

Roughness zones

Figure S2 shows the spatial distribution of the 11 distinct roughness zones with similar grain characteristics.

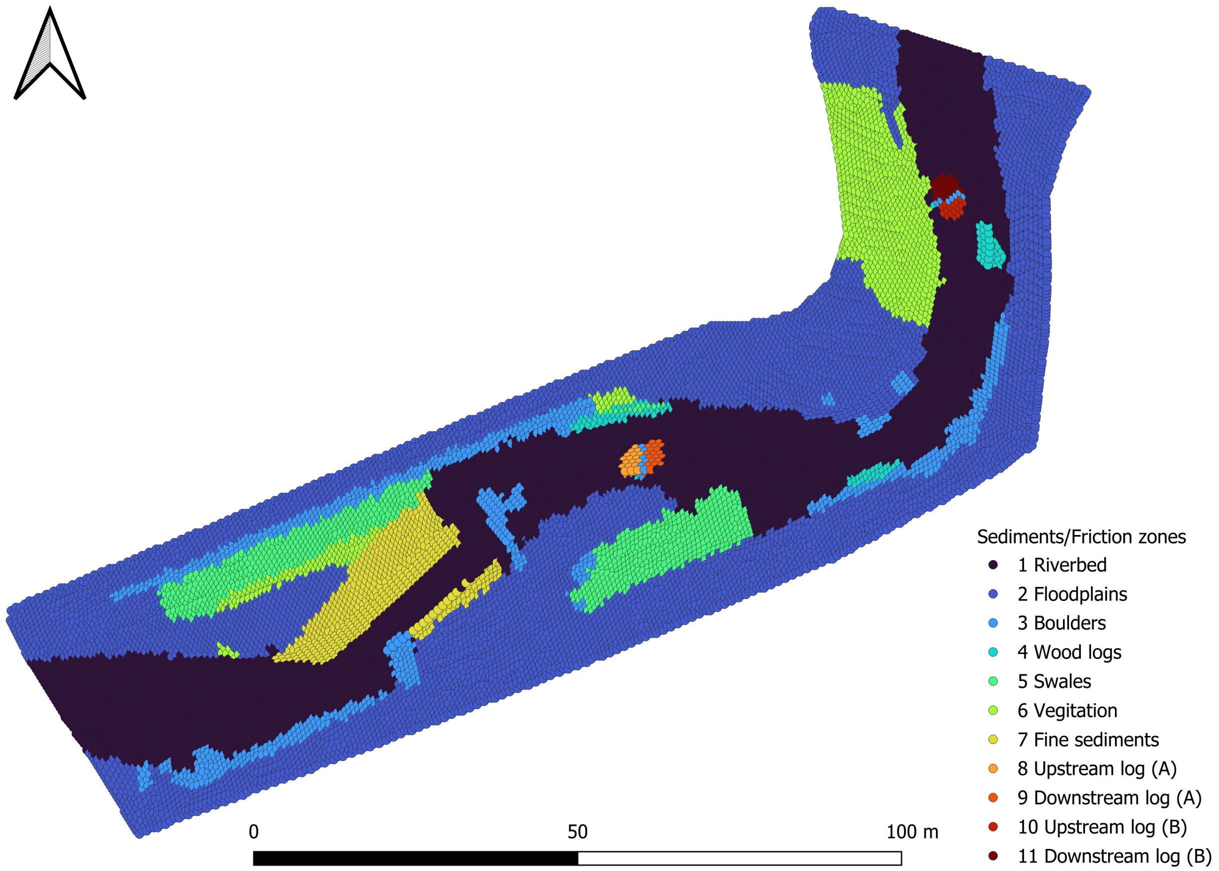

Figure S2. Spatial distribution of the 11 roughness zones.

Vegetation and the Baptist formula

Vegetation-induced friction was calculated according to the formula from Baptist *et al.*[12] (Eq. 8), which accounts for both submerged and emergent vegetation. This formulation considers vegetation as an additional hydraulic resistance element, with its effect depending on stem density, submergence, and flow conditions. The friction factor f" was determined as:

$$f'' = \begin{cases} 4C_D \cdot \frac{D\,h}{\Delta^2} & h \leq h_p \\ 4 \cdot \left(\frac{1}{\sqrt{C_D \frac{Dh_p}{\Delta^2}}} + \frac{1}{\sqrt{2}\kappa} \ln \frac{h}{h_p} \right)^{-2} & h > h_p \end{cases} \tag{8}$$

where C_D is the drag coefficient, D is the vegetation stem diameter, h is the water depth, h_P is the height of vegetation, Δ is the horizontal distance between vegetation, and κ is the Von Kármán constant[13], equal to 0.41.

The vegetation parameters required for the application of Equation 8 were derived from ortho-imagery, on-site photography, and field observations as follows:

- Vegetation height h_P: 0.4 m, based on the observed height of vegetation on the field.
- Stem characteristics: cylindrical stems with a diameter of D = 0.055, and a horizontal spacing of Δ = 0.1 m.
- Bulk drag coefficient C_D: assumed to be 1, following the original formulation of Baptist *et al.*[12].

While the assumption of C_D = 1 follows the original formulation of Baptist *et al.*[12] and is widely applied in studies on flow through rigid cylindrical vegetation, it introduces certain limitations. First, it does not account for the flexibility of natural vegetation, which can reconfigure under high-flow conditions, reducing drag through streamlining[14]. This may lead to an overestimation of vegetation-induced resistance, particularly in strong currents. Second, the drag coefficient depends on the Reynolds number ($Re = \frac{UD}{\nu}$), meaning that in turbulent river environments, C_D can vary with increasing Re, whereas a fixed value does not capture this effect[15]. Additionally, vegetation density influences wake interactions. In densely vegetated areas, sheltering effects reduce drag, while in sparse regions, individual stems experience higher resistance[16]. The assumption of C_D = 1 does not explicitly account for these density-dependent variations. Furthermore, while the formulation distinguishes between submerged and emergent vegetation, it does not fully capture the influence of flow depth on drag, as experimental studies have shown that submergence alters wake dynamics and flow-vegetation interactions, potentially affecting the overall drag force[17]. Despite these limitations, adopting C_D = 1 remains a consistent approach for estimating vegetation-induced roughness.

The roughness coefficient at the inlet and outlet walls was back-calculated as Manning's n = 0.025 m$^{-1/3}$ s using the Manning-Strickler equation[18,19] and acoustic Doppler current profiler (ADCP) cross-sectional measurements, assuming steady, uniform flow conditions at the time of the measurement:

$$u = \frac{1}{n} R_{hy}^{\frac{2}{3}} S^{\frac{1}{2}} \tag{9}$$

ADCP and unmanned aerial vehicle (UAV) measurements provided the following hydraulic parameters:

- Inflow boundary bottom elevation: 329.540 m a.s.l.
- Outflow boundary bottom elevation: 328.872 m a.s.l.
- Elevation difference: 0.668 m
- Channel slope: 0.00294
- Wetted cross-sectional area: 7.697 m^2

- Wetted perimeter: 14.44 m
- Mean flow velocity: 1.339 m s^{-1}

SI 4 Calibration procedure

The iterative model optimization (calibration) relied on Root Mean Square Error (RMSE) calculations to refine roughness heights based on the agreement between simulated and observed values. The optimization workflow began with an initial steady-discharge (2 m^3 s^{-1}) model run using preliminary estimates for the roughness heights, which were derived from prior studies[20] and known values for similar morphological patterns and sediment sizes.

Following the initial model run, RMSE was computed by comparing simulated and observed flow velocity and water depth. Based on the RMSE results, adjustments were made to the roughness heights to improve the simulation outcomes. Specifically, this iterative process involved systematically modifying the roughness heights, recalculating RMSE after each model run, and analyzing the impact of the changes on the accuracy of the simulated results.

The iterative roughness optimization continued until RMSE was minimized, signifying that the simulated flow velocity and water depth were in close agreement with the field measurements. Once a satisfactory RMSE value was obtained for steady flow conditions, the final optimized roughness heights were retained for further quasi-steady (unsteady) simulations.

Water depth and velocity values from the simulation were extracted at the end of the artificial flood, once the flow had returned to a baseflow of 2 m^3 s^{-1} at locations where field measurements were available. Comparisons were made between the modeled water depths and flow velocities and the field measurements, with the linear trends visualized for clarity.

Figure S3 shows the water depth point values at log A and log B, respectively. The measured values are plotted on the x-axis whereas the modeled water depths are shown on the y-axis.

Figure S4 shows the flow velocity point values at log A and log B, respectively. The measured values are plotted on the x-axis whereas the flow velocities obtained from the numerical simulation are shown on the y-axis.

A similar iterative calibration procedure as for hydraulics was applied to the Shields parameter for the grain diameter classes so that grains did not move at 2 m^3 s^{-1}. The preliminary estimate for the Shields parameter was 0.047. The optimization process followed the same steps as for the roughness heights, that is, an initial model run, RMSE computation, Shields parameter adjustments, and iterative refinement until RMSE was minimized. The final optimized Shields parameter values were then used in subsequent quasi-steady (unsteady) simulations.

SI 5 Digital elevation model (DEM) of Differences (DoD)

The morphological changes observed in the study area were assessed by analyzing elevation differences between pre- and post-flood terrain data. These differences highlight distinct zones of erosion and deposition, that is, topographic change. For this purpose, a DoD was generated by subtracting pre-flood from post-flood elevation on a pixel-by-pixel basis. To account for measurement uncertainty due to grain protrusion, only elevation changes exceeding ± 0.1 m were considered. The DoD was computed using the QGIS[21] Raster Calculator with the following expression:

```
    if ( "bottom-tend-highres@1" - "bottom-t000-highres@1" >= 0.1 ),
         "bottom-tend-highres@1" - "bottom-t000-highres@1" - 0.1,
    else if ( "bottom-tend-highres@1" - "bottom-t000-highres@1" <= -0.1 ),
              "bottom-tend-highres@1" - "bottom-t000-highres@1" + 0.1,   0.0
```

In this expression, `"bottom-tend-highres@1"` and `"bottom-t000-highres@1"` represent the post- and pre-flood elevation rasters, respectively. The difference between the rasters gives the topographic change at each pixel. To exclude variations within the grain size range, values above 0.1 m (deposition) were adjusted by subtracting 0.1 m, while values below -0.1 m (erosion) are adjusted by adding 0.1 m. Changes between -0.1 m and +0.1 m were set to zero to filter out grain size

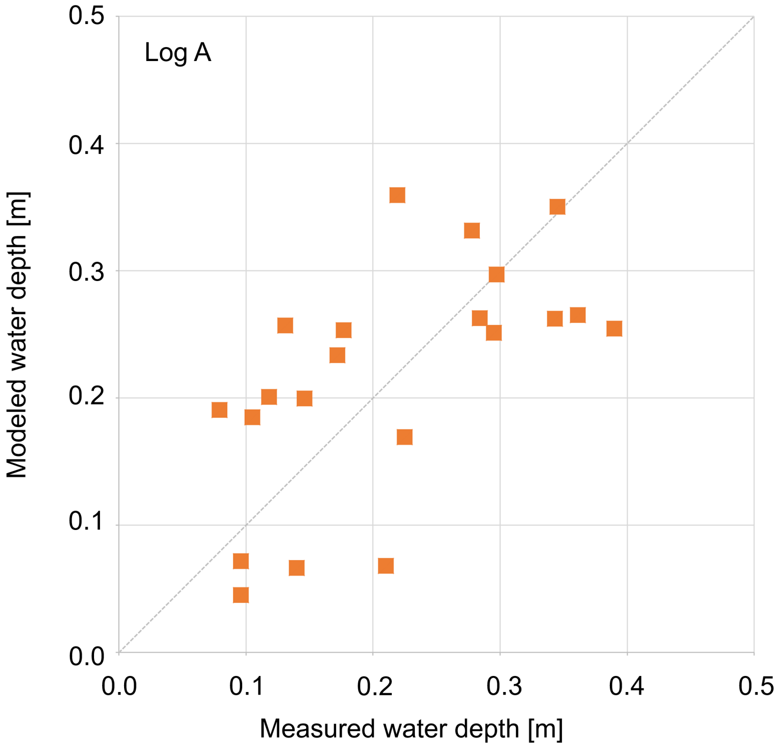

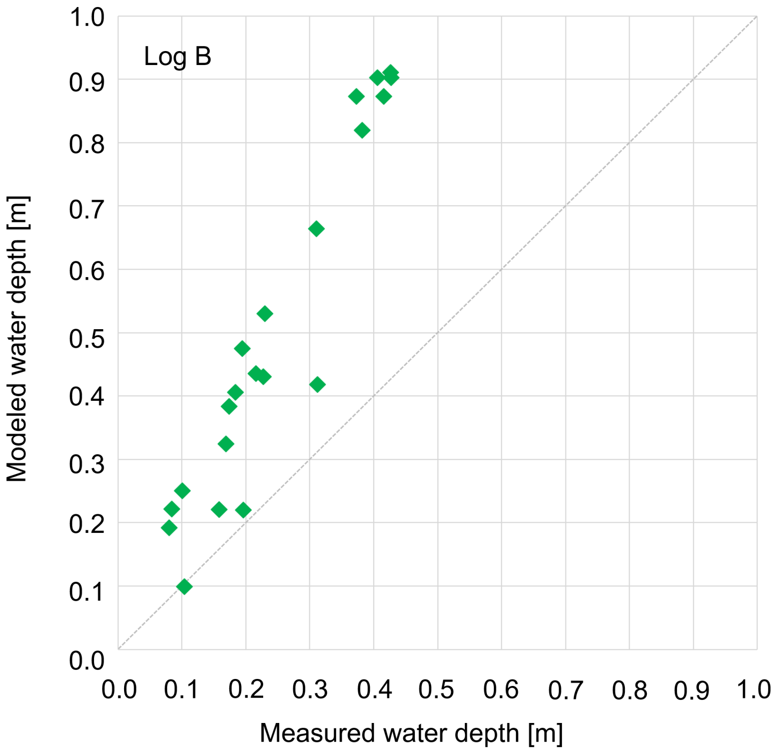

Figure S3. Modeled and measured water depths at log A and log B.

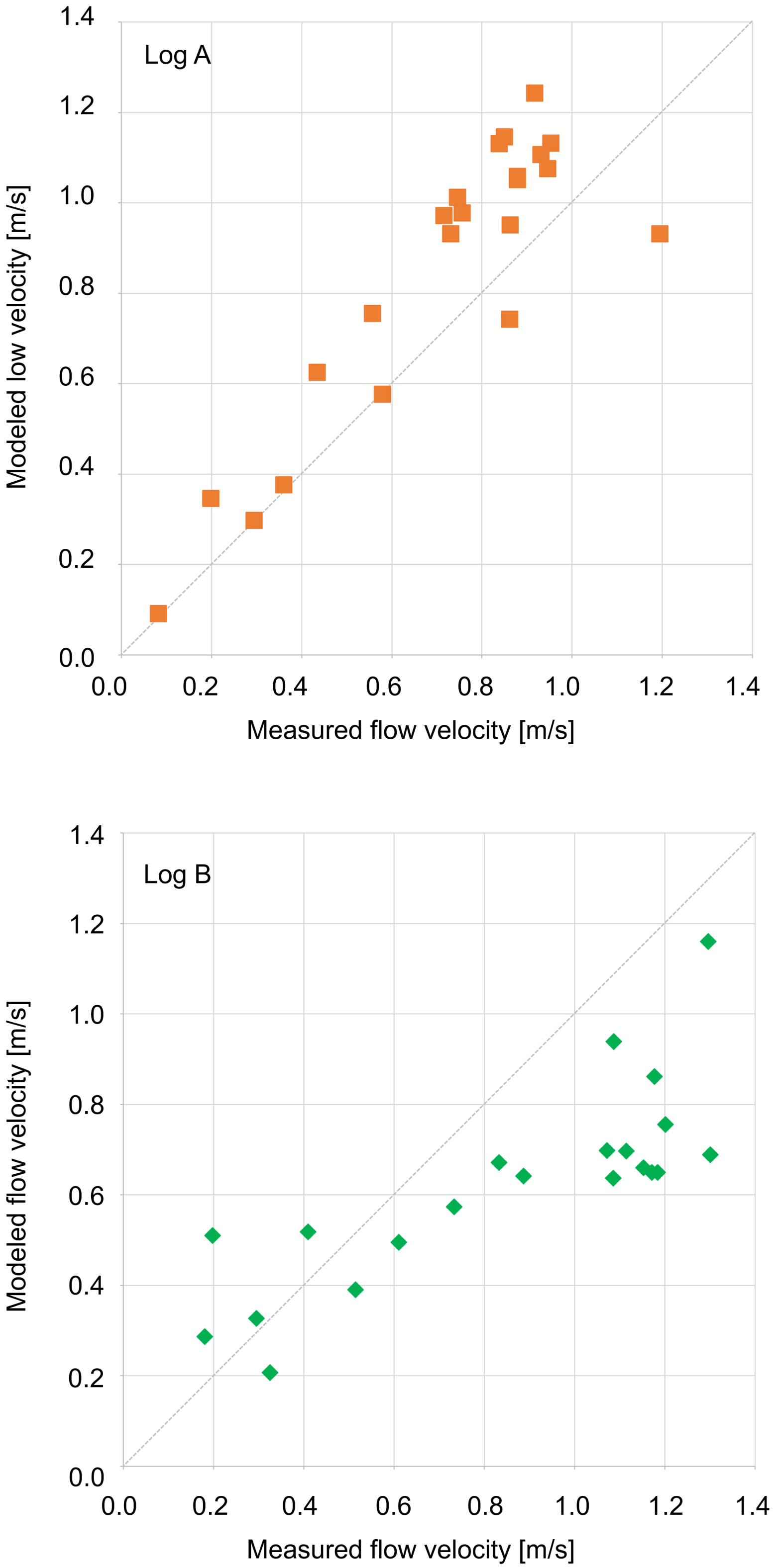

Figure S4. Modeled and measured flow velocities at log A and log B.

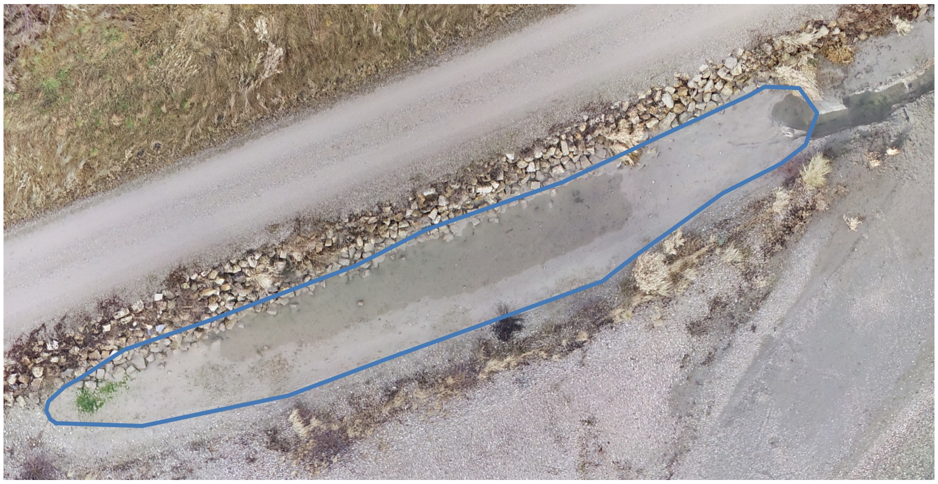

Figure S5. Drone image of a swale in the riverbed, characterized by fine sediment deposits. Source: IWS (2022).

noise. This treatment ensured that only meaningful topographic changes were considered, with negative DoD values indicating erosion and positive values representing deposition.

Additional figures

References

1. Smolders, S. How a flow aligned mesh improves TELEMAC model results. In Bourban, S. E. *et al.* (eds.) *Proceedings of the Xxviiith TELEMAC User Conference 18-19 October 2022*, 49 (EDF R&D, Paris-Saclay, France, 2022).

2. NRC-CNRC. Blue kenue 3.12.0-alpha - reference manual. National Research Council Canada (2020).

3. van Rijn, L. C. Sediment Transport, Part II: Suspended Load Transport. *J. Hydraul. Eng.* **110**, 1613–1641, DOI: 10.1061/(ASCE)0733-9429(1984)110:11(1613) (1984).

4. Meyer-Peter, E. & Müller, R. Formulas for Bed-Load transport. In *IAHSR, Appendix 2*, vol. 2nd meeting, 39–65 (1948).

5. Wong, M. & Parker, G. Reanalysis and Correction of Bed-Load Relation of Meyer-Peter and Müller Using Their Own Database. *J. Hydraul. Eng.* **132**, 1159–1168, DOI: 10.1061/(ASCE)0733-9429(2006)132:11(1159) (2006).

6. Exner, F. M. Über die Wechselwirkung zwischen Wasser und Geschiebe in Flüssen [About the Interdependency of Water and Bed load in Rivers]. *Akademie der Wissenschaften Wien, math.-naturw. Klasse, Sitzungsberichte, Abt. IIa* **134**, 165–203 (1925).

7. Hirano, M. River-Bed Degradation with Armoring. *Proc. Jpn. Soc. Civ. Eng.* **1971**, 55–65, DOI: 10.2208/jscej1969.1971.195_55 (1971).

8. Blom, A. Different approaches to handling vertical and streamwise sorting in modeling river morphodynamics. *Water Resour. Res.* **44**, DOI: 10.1029/2006WR005474 (2008).

9. Stecca, G., Siviglia, A. & Blom, A. An accurate numerical solution to the Saint-Venant-Hirano model for mixed-sediment morphodynamics in rivers. *Adv. Water Resour.* **93**, 39–61, DOI: 10.1016/j.advwatres.2015.05.022 (2016).

10. Tassi, P. *et al.* GAIA - a unified framework for sediment transport and bed evolution in rivers, coastal seas and transitional waters in the TELEMAC-MASCARET modelling system. *Environ. Model. & Softw.* **159**, 105544, DOI: 10.1016/j.envsoft.2022.105544 (2023).

11. Shields, A. *Anwendung Der Ähnlichkeitsmechanik Und Der Turbulenzforschung Auf Die Geschiebebewegung [Application of the Similarity in Mechanics and Turbulence Research on the Mobility of Bed Load]*, vol. 26 (Preußische Versuchsanstalt für Wasserbau und Schiffbau, Berlin, Germany, 1936).

12. Baptist, M. *et al.* On inducing equations for vegetation resistance. *J. Hydraul. Res.* **45**, 435–450, DOI: 10.1080/00221686.2007.9521778 (2007).

13. Von Kármán, T. Mechanische Ähnlichkeit und Turbulenz [Mechanical similarity and turbulence]. In *Third International Congress for Applied Mechanics*, vol. 1 of *International Congress for Applied Mechanics*, 79–93 (Gesellschaft der Wissenschaften zu Göttingen, Stockholm, Sweden, 1930).

14. Aberle, J. & Järvelä, J. Flow resistance of emergent rigid and flexible floodplain vegetation. *J. Hydraul. Res.* **51**, 33–45, DOI: 10.1080/00221686.2012.754795 (2013).

15. Lee, J. K., Roig, L. C., Jenter, H. L. & Visser, H. M. Drag coefficients for modeling flow through emergent vegetation in the Florida Everglades. *Ecol. Eng.* **22**, 237–248, DOI: 10.1016/j.ecoleng.2004.05.001 (2004).

16. Nepf, H. M. Drag, turbulence, and diffusion in flow through emergent vegetation. *Water Resour. Res.* **35**, 479–489, DOI: 10.1029/1998WR900069 (1999).

17. Wu, F.-C., Shen, H. W. & Chou, Y.-J. Variation of Roughness Coefficients for Unsubmerged and Submerged Vegetation. *J. Hydraul. Eng.* **125**, 934–942, DOI: 10.1061/(ASCE)0733-9429(1999)125:9(934) (1999).

18. Manning, R. On the flow of water in open channels and pipes. In *Transactions of the Institution of Civil Engineers of Ireland*, vol. 20, 161–207 (Civil Engineers of Ireland, 1891).

19. Strickler, A. Beiträge zur Frage der Geschwindigkeitsformel und der Rauhigkeitszahlen für Ströme, Kanäle und geschlossene Leitungen [Contributions to the question of the velocity formula and the roughness figures for streams, channels and closed pipes]. *Mitteilungen des Eidgenössischen Amtes für Wasserwirtschaft, Switz.* **16**, 357 (1923).

20. Schwindt, S. *et al.* Fuzzy-logic indicators for riverbed de-clogging suggest ecological benefits of large wood. *Ecol. Indic.* **155**, 111045, DOI: 10.1016/j.ecolind.2023.111045 (2023).

21. QGIS Development Team. QGIS. Open Source Geospatial Foundation Project (2022).
